# Perspectives from law enforcement officers who respond to overdose calls for service and administer naloxone

**DOI:** 10.1186/s40352-022-00172-y

**Published:** 2022-02-25

**Authors:** Hope M. Smiley-McDonald, Peyton R. Attaway, Nicholas J. Richardson, Peter J. Davidson, Alex H. Kral

**Affiliations:** 1 Division for Applied Justice Research, RTI International, Research Triangle Park, North Carolina, USA; 2 Department of Medicine, Division Global Public Health, University of California, San Diego, La Jolla, California, USA; 3 Community Health Research Division, RTI International, Berkeley, California, USA

**Keywords:** Law enforcement, Police, Naloxone, Overdose, On scene overdose response

## Abstract

**Background:**

Many law enforcement agencies across the United States equip their officers with the life-saving drug naloxone to reverse the effects of an opioid overdose. Although officers can be effectively trained to administer naloxone, and hundreds of law enforcement agencies carry naloxone to reverse overdoses, little is known about what happens on scene during an overdose call for service from an officer’s perspective, including what officers perceive their duties and responsibilities to be as the incident evolves.

**Methods:**

The qualitative study examined officers’ experiences with overdose response, their perceived roles, and what happens on scene before, during, and after an overdose incident. In-person interviews were conducted with 17 officers in four diverse law enforcement agencies in the United States between January and May 2020.

**Results:**

Following an overdose, the officers described that overdose victims are required to go to a hospital or they are taken to jail. Officers also described their duties on scene during and after naloxone administration, including searching the belongings of the person who overdosed and seizing any drug paraphernalia.

**Conclusion:**

These findings point to a pressing need for rethinking standard operating procedures for law enforcement in these situations so that the intentions of Good Samaritan Laws are upheld and people get the assistance they need without being deterred from asking for future help.

## Background

Over 700,000 people died in the United States (U.S.) from drug overdoses between 1999 and 2017, with opioids being involved in 70% of drug overdose deaths in 2018 (Wilson et al.*,*^2020^). Recent provisional data from the Centers for Disease Control and Prevention (CDC), 2019 show that there were an estimated 100,306 reported overdoses during the12-month period ending in April 2021 (Ahmad et al., 2021). As the opioid overdose mortality crisis continues, attention has focused on how law enforcement can save individuals who are overdosing (Lurigio et al.*,*^2018^; Police Executive Research Forum (PERF), 2016) by equipping officers with the overdose reversal drug naloxone (Carroll et al.*,*^2020^; Police Executive Research Forum (PERF), 2017; White et al.*,*^2021^). The Food and Drug Administration approved naloxone for treating opioid overdoses in 1971. Naloxone is a safe opioid antagonist medication that can rapidly reverse an opioid overdose. Because it is safe and effective at reversing overdoses and has no potential for abuse, states have passed laws facilitating lay person access and the federal government has called for increased access to all approved forms of naloxone (Health and Human Services, 2018; Food and Drug Administration, 2019). Although paramedics have carried naloxone since the 1980s, it has only been in the past decade that law enforcement agencies have begun training officers to administer naloxone. Law enforcement use of naloxone—most commonly in an intranasal spray form, trade name Narcan—has been associated with a decrease in opioid overdose deaths (Rando et al.*,*^2015^), can facilitate access to this needed resource in some rural areas (Bennett and Elliott, 2021), and has been determined to be cost effective (Townsend et al.*,*^2020^). The North Carolina Harm Reduction Coalition (NCHRC) (2021) estimates that over 1200 agencies in the U.S. equip officers with naloxone.

Early research on law enforcement officer naloxone administration highlighted the feasibility of equipping officers with naloxone (Fisher et al.*,*^2016^; Saucier et al.*,*^2016^), noting that although officers receiving training were proficient in identifying and administering naloxone to overdose victims, equipping officers requires funding, inner agency communication, and consistent training (Kitch and Portela 2016). Other studies have found that officers are generally positive towards naloxone training and administering naloxone following an overdose (Carroll et al.*,*^2020^; Green et al.*,*^2013^; Purviance et al.*,*^2017^; Ray et al.*,*^2015^; Wagner et al.*,*^2016^; White et al.*,*^2021^).

Although officers can be effectively trained to administer naloxone, recent research has documented the emotional toll of handling overdose calls for service, including compassion fatigue or burnout (Carroll et al.*,*^2020^). Research has also documented concerns among first responders that overdose prevention strategies enable continued drug use (Bessen et al.*,*^2019^; Kilwein et al.*,*^2019^; Saunders et al.*,*^2019^; White et al.*,*^2021^; Winograd et al.*,*^2020^). Berardi et al. (2021) found that whether Canadian officers administered naloxone at the scene of an overdose depended on: 1) having sufficient knowledge and concern about whether fentanyl was present, 2) being knowledgeable about naloxone and trained in its use, 3) the medication being readily available to officers, and 4) being willing to administer it to the public. In a study of Tempe, Arizona patrol officers equipped with naloxone, White et al. (2021) noted differences by sex, race, education, length of service, and survey wave regarding officers’ attitudes towards people who use drugs, risk compensation beliefs, and attitudes towards overdoses.

Officers responding to overdose related calls for service are governed by Good Samaritan Laws (GSLs). Most states have GSLs which protect overdose victims and those calling 911 for overdose response from arrest and prosecution (Mauri et al.*,*^2020^; National Conference of State Legislatures, 2019). Yet GLSs vary in their protections, with most providing protection to the person who requested emergency responders from prosecution for minor drug possession and nearly half protecting the caller from being arrested for those crimes (Davis and Carr, 2015; PDAPS, 2018). States also vary with respect to their protections from prosecution for drug paraphernalia, probation or parole violations, and whether reporting an overdose is considered a mitigating factor for sentencing (Davis and Carr, 2015; PDAPS, 2018). Recent work in this area shows that GSLs that protect callers and victims from arrest for these types of offenses may be more effective in reducing fear about contacting emergency services than those GSLs with protections from charges/prosecution (Hamilton et al.*,*^2021^). One study found that after states enact a GSL, they have 15% reductions in opioid overdose deaths (McClellan et al.*,*^2018^). Other studies demonstrate that people who use drugs are unaware of their state’s GSLs and their associated protections (e.g., Fadanelli et al.*,*^2020^; Schneider et al.*,*^2020^).

Although officers have been found to be aware of their state’s GSL (Banta-Green et al.*,*^2013^; Carroll et al.*,*^2020^; Saucier et al.*,*^2016^; Wagner et al.*,*^2016^), research has shown that overdose calls for service can result in arrests (Lowder et al.*,*^2020^) for crimes including public intoxication, possession of drug paraphernalia, or outstanding warrants (Deonarine et al.*,*^2016^). Carroll et al.’s (2020) sample of 2829 officers in 20 states found that 37% had reported administering naloxone at least once in the past 6 months and 36% reported having made at least one arrest at an overdose scene. Arrests at an overdose scene may deter community members from calling 911 in the future, as they may be concerned they will end up arrested or in jail (Wagner et al.*,*^2019^). Reducing community members’ trust in the 911 system could lead to overdose deaths that could otherwise been prevented.

Missing from this body of work is an accounting of what occurs on scene during an overdose call for service from an officer’s perspective, including how officers experience the overdose incident and what officers perceive their duties and responsibilities to be as the incident evolves. This study, which includes qualitative findings from interviews with officers who have responded to overdose calls for service across four U.S. jurisdictions, fills an important research gap about what happens on-scene before, during, and after officers administer naloxone to people who are suspected to be overdosing.

## Methods

The present analysis draws from a larger mixed method study examining law enforcement agency (LEA) naloxone programs in the U.S. This Arnold Ventures-supported study was designed to examine services, policies, practices, operations, and resources of LEA naloxone programs in the U.S. The overall study was reviewed and approved by RTI International’s Institutional Review Board. We used an inductive qualitative analysis approach described by Thomas (2006) to understand the experience and process of officers administering naloxone to people who overdose.

### Recruitment

Five LEAs were chosen for the qualitative study component to represent a diverse set of agencies based on community characteristics (i.e., population size, geography, overdose mortality burden), agency-level characteristics (i.e., number of sworn officers), and overdose response characteristics (i.e., presence and maturity of a naloxone program, key partnerships with other first responders and treatment providers) (Bagley et al.*,*^2019^). Site 4 was chosen because the LEA leadership opted to not equip its officers with naloxone, despite the area’s well-documented opioid overdose problem. One LEA declined to participate, and one LEA was unable to participate because of scheduling difficulties. In both cases, a neighbouring community was chosen as a suitable substitute and the LEA serving in those jurisdictions agreed to participate.

The team, which included the Police Executive Research Forum as a study partner, contacted the police chief in each of the five proposed sites by email with an invitation to participate in the study. The introductory e-mail included a brief study description, the types of questions that would be asked, and confirmation about the site’s naloxone program. Follow-up telephone calls were placed 3–4 business days later and site visit logistics were coordinated, usually with an LEA captain or other manager who oversaw the LEA naloxone program. The LEA site lead identified the officers who participated in the interviews.

To maintain confidentiality of the LEAs, we do not provide the names of the agencies, rather we provide an overview of the site characteristics, including their geographic location, jurisdiction size, and the year the naloxone program started (Table 1). No officers were interviewed in Site 5, located in the East, due to complications associated with gathering data during the COVID-19 pandemic. Thus, the data used for the purposes of this study come from Sites 1, 2, 3, and 4.

**Table 1:** Law Enforcement Agency Site Characteristics

Site	Census Region	Jurisdiction Population as of 2018 Census	Year Law Enforcement Naloxone Program Started
Site 1	West	> 1,000,000	2017
Site 2	Midwest	250,000-999,999	2016
Site 3	South	25,000-249,000	2015
Site 4	South	< 25,000	No naloxone program

### Participants

The individuals interviewed for the qualitative study included law enforcement officers who have administered naloxone at least once to someone they believed to be overdosing (*n* = 12) and officers who have responded to an overdose but have not administered naloxone (*n* = 5). Most of the officers were Caucasian (*n* = 9) and male (*n* = 13). The number of years of law enforcement experience ranged from 2 to 22 years.

### Data collection

Data were collected between January and May 2020. Interviews were conducted in-person on site at LEA sites 1–4 by three-person study teams, consisting of a lead interviewer, a co-lead, and a note taker. In all cases, author Attaway was the note taker, with lead and co-lead interviewer roles being rotated between authors [Smiley-McDonald, Kral, and Davidson]. Lead and co-lead interviewers also made their own field notes during and immediately after each interview. Informed consent was obtained before interviews were conducted, and all interviews were recorded following the consent of all participants. There were no participant refusals.

After each site visit, a set of notes for each interview was prepared by author Attaway, in which the recording from each interview was listened to in full alongside written notes, and an ‘interview notes’ file prepared comprising of a combination of summary notes and direct transcription of illustrative comments made. All site visit team members then reviewed these interview notes and added additional comments based on their own field notes. No identifying information about the respondents was retained. Each respondent was given a numeric identifier to keep the records distinct and confidential.

### Qualitative study guide

Interviews followed a semi-structured qualitative study guide developed by the study team to explore the following domains: information related to the community context that influenced naloxone program implementation, LEA characteristics, naloxone program attributes, oversight, outreach efforts, community partnerships, and officers’ experiences responding to overdose incidents. The guide was then iteratively modified as interviews progressed to allow issues emerging in earlier interviews to be explored in more detail in later interviews.

### Analysis

Analysis followed an iterative inductive approach based on that described by Thomas (2006). Specifically, following each site visit, participating team members 1) read through the interview notes from that visit, and 2) participated in a debriefing meeting to discuss initial impressions, identify emerging themes, and possible issues to follow up on in subsequent interviews. As data collection drew to an end, no new themes emerged. Site debriefing notes were used to construct a master list of codes that represented topics addressed by specific questions within the interviews and emergent themes, issues, and concepts. The master code list was created before the interview notes were coded and included definitions where appropriate (Nowell et al.*,*^2017^).

At the end of data collection, all team members who had been involved in site visits and interviews then participated in a series of analytic meetings to review, discuss, and refine codes and themes which had emerged during data collection. These codes were then used by author Attaway to code all interview notes using NVivo 12.0 (QSR International, 2021). Coded data were verified by author Smiley-McDonald before they were finalized. Finally, coded data were reviewed by the team to ensure all relevant themes had been described, and to articulate patterns and connections across themes.

## Results

First, we present context for overdose calls for service in the officers’ communities and their experience using naloxone. The rest of the findings are presented temporally by the sequence of events for how overdose calls are handled by law enforcement, starting with the call for service and ending with the incident resolution.

### Context for overdoses and law enforcement naloxone use

Some of the officers interviewed described overdose calls for service as coming in waves. As one officer noted, ‘Sometimes we will go a few weeks without responding to any overdoses and then we will respond to several in one week. It just depends on the market.’ Another officer noted that:

There are definitely more overdose calls on nights, holidays, and during more depression heavy times. For people who are already depressed, these times make it more intense and they need more drugs to compensate and so they tend to get a hotter batch or keep using until they feel numb.

Among the officers who provided an estimate for the number of times they administered naloxone, it ranged from once to ‘between 100-200 times.’ The officer with the highest number of administrations noted that ‘you definitely get the sense from the department that they want you to use it [naloxone] every time.’

Jurisdictions had different policies for where naloxone was kept for easy access. One jurisdiction provided a tool belt pouch for naloxone kits so that officers could carry it on their person. Some jurisdictions had policies that the naloxone kits be stored in officer first aid kits or ‘shotgun bags,’ which were described as being located on the passenger side of their car or in glove compartments. It was noted that uniform storage practices were important in case an officer had to access another officer’s car to retrieve more naloxone.

### Calls for overdose response

Nearly all officers noted that law enforcement arrive on the scene first because, as one officer observed, ‘we are always mobile in our cars.’ An officer who worked a square mile neighborhood said that it was not uncommon to beat the EMS team by 3–5 min. Thus, he uses naloxone to ‘buy the victim more time.’ Conversely, in the smaller jurisdiction, it was noted that law enforcement may arrive only seconds ahead of EMS and the Fire Department. As one officer in that jurisdiction said:

There was never a time that [I] would have been able to administer Narcan before the EMS got there. When [I get] on scene [I] will go through the scene, check for a pulse, and report back on the radio what’s happening.

### Securing the scene

Many officers described putting on disposable medical gloves while in their car en route to an overdose scene or as soon as they arrive as part of their standard precautionary preparations. Upon arrival, some officers noted that they first make sure that the scene is ‘secured.’ One officer noted that for overdoses, his department treats these calls as a medical call for service as opposed to a potential crime scene. Thus, for an overdose, ‘scene security is not as locked down.’ However, this officer also noted that if it is known that a death has occurred, they will lock the scene down and treat it like a homicide call for service.

Many officers said that it is rare to find drugs or paraphernalia on scene because bystanders—including those who called 911—usually clear away such items prior to their arrival. Officers from the urban jurisdictions typically reported overdose victims being found in cars, alleys, gas station or fast-food restaurant bathrooms, or homes. Officers from the small jurisdiction indicated that overdose victims were commonly found in cars or outside in the front yards of residences. In this jurisdiction, people who overdose are often dragged to the front yards of homes for two reasons: 1) it prevents police from entering the residence and 2) overdose victims cannot be arrested for public intoxication if they are within private property boundaries.

Some officers described that they often find overdose victims alone upon arrival. In these cases, officers presume that whoever called 911 made sure that help was on the way before they left the victim to avoid getting into trouble with the police. Other officers described how they handle crowd control when bystanders are present. One officer elaborated:

When you’re responding to someone for a medical emergency you never know who the bystanders are. It could be family members or close friends and if they are, I would assume they may stand around in that area to kind of watch over them to make sure their loved one is safe. In those situations, crowd control is very important and I think our officers do a really good job at understanding the people around them so when we are called to an emergency we will request an extra unit to do crowd control. Just being able to vocalize that we are there to help them normally prompts them to give us the space we need.

It was noted that bystanders are often upset because they may have tried CPR or other measures that have not worked. Another officer provided more detail about how they handle bystanders considering the possible ways they can influence the revived overdose victim’s decision-making. They typically ask bystanders what and how much drugs the person consumed. The officer elaborated:

When I’m on scene it is about that subject. I want them to be healthy. I want them to come back to life, and I raise my voice a lot, I tell them [bystanders] to get back. Because my main concern at this point is not them, she needs us … I tell all bystanders to stand at the end of the road because I don’t want them to interfere with the individual’s decision. I don’t know how they know her. If they are a friend, or if they are supplying [the drugs], so I want them to be separated so that way she can make her own decision, and feel free to make her own decision and not be influenced.

Finally, officers serving communities where naloxone is widely distributed by the health department or other human social service agencies noted that it was common for lay people to have already administered naloxone prior to law enforcement’s arrival.

### Administering naloxone to overdose victims

Overdose procedures in the jurisdiction where the law enforcement agency did not equip their officers with naloxone were like those in the other jurisdictions before they implemented naloxone programs. Below is a quote about this process from an officer in the largest urban jurisdiction:

Before [I had naloxone] I had to do the old traditional method of either turning them on their side or doing a sternum rub or calling out to them just to stimulate them even if it makes them uncomfortable, just to keep them around long enough until the paramedics came or even [administer] chest compressions. I’ve never had to do CPR.

Prior to being equipped with naloxone, one officer told a story about how helpless she felt upon arriving to a scene that involved an adult who had overdosed during a child’s birthday party. She described feeling ‘desperate and weird’ because although someone called for help, she could only stand over the person and wait for the paramedics.

Officer discretion was also described about the procedures used upon arriving on scene. One officer noted that he “normally goes straight to Narcan without doing [a] sternum rub.” Another said they do not administer naloxone if they see that the victim is breathing on their own since officers have been told to only give it to suspected overdose victims if they are not breathing.

Officers who had administered naloxone provided very detailed accounts of times when they revived a person who was overdosing. As one officer observed, administering naloxone can be ‘nerve-wracking because you have someone’s life in your hands.’ An example of one account is below:

We saw the syringe and the little tin cap, and he [the victim] was out. You could tell his breathing was labored like his body was trying to breathe but he was having a hard time … I remember in my mind thinking just push the button, put it in his nostril, hold the other closed, and push it in. Try to time the breaths, and I did it about three times. Because I didn’t remember exactly how many times you were supposed to do it, but I do remember that they said its safe if you do it multiple times. It’s okay, it won’t harm anybody. I’ve seen it administered by the fire department and heard that right when you administer, they [officer snapped fingers] instantly wake up. He wasn’t waking up so I gave him a third, and after that third [dose] one he just stood right up.

In administering naloxone, officers often elaborated on how they would see the victim go from greyish blue or purple and then following a naloxone administration, being revived seconds later with a gasping breath and changed pallor. As one officer elaborated, ‘Usually after 30 seconds you can see their color come back because they go from a purple blue to a normal skin color and then they just wake up.’

Across the agencies that equip their officers with naloxone, another common theme was that officers described administering multiple doses of naloxone to suspected overdose victims, particularly since, as one officer put it, ‘you aren’t going to OD someone with Narcan.’ Naloxone was also described as a finite resource as officers were typically carrying only 1–2 doses on their person or in their cars. As one officer noted:

It’s a weird feeling because they only have one dose and there is nothing you can do until fire or EMS comes. I would like to have more than one dose.

### Post naloxone administration

Several themes emerged when officers described what typically happens when an overdose victim is revived. Some described it from the victim’s perspective:

I have seen people come out violent a few times, but not every time. I think it’s a reaction out of fear and not understanding what’s going on. Everyone has the fight or flight mentality … I don’t know why it happens but my best guess would be that if the last thing you remember is going into the bathroom and then you wake up with people standing around you and crowding you, that reaction comes from fear and not understanding what is going on around you.

The theme of people coming out of an overdose and being angry or violent was brought up in nearly all interviews, with either officers saying that it was at least somewhat common while other officers dismissed the notion of angry or violent reactions as atypical. Among officers that said it was at least somewhat common, an example quote is as follows:

I have had a few people get legitimately upset with you because you ruined their high. I try to stay close because some people do come out swinging. I had one grab a medic and I had to kick them in the face.

Other officers dispelled the notion that violent reactions are typical:

There’s this big concern [the revived overdose victims] just instantly, Hulk-smash, turn green, and go nuts. I have not personally seen that kind of extreme [reaction].

Officers provided context for an overdose victim’s immediate reactions as being motivated to not get into trouble with law enforcement. Some officers also described how the rapid revival of an unconscious person was jarring to them and how they treated the person before and after they gained consciousness.

We call them zombies sometimes because they are dead, there is no pulse, and then we give them Narcan and we will be sitting there and 3–4 min later they will wake up totally,’ Hey what’s up what are you guys doing here?’ … The first couple of times you do it it’s weird because this guy wasn’t breathing five minutes ago. It’s not like a drunk where you wake them up and they are out of it. They are totally back to normal. It’s off-putting. The most common reaction is that they play it off like they were sleeping. I’m like, dude, I was kicking you in the ribs. You were not tired.

### Incident resolution

Officers were asked what happens after the overdose victim is revived. In two of the jurisdictions, victims were told they had to be transferred to the hospital, or they would be taken to jail. Officers’ understanding of the reasons for these practices included the risk that people would slip back into overdose again:

It’s not an “If”—it’s a ‘You’re going to the hospital.’ If they won’t go, we will force them to go under immediate detention. They have to meet certain criteria and the medics will tell us that if they took a lot of heroin or required several doses of Narcan to wake up. If they don’t go to the hospital and get more Narcan they will overdose again. We use that as a justification of making them go.

Officers from these two jurisdictions voiced some opinions about the policies that force overdose victims to go to the hospital and how the local EMS plays a part in enforcing this practice.

Our department is really good. We don’t let people walk away that have overdosed. They will go to the hospital whether it’s on their own or through immediate detention. Because once you walk off, that Narcan can wear off and you will drop off. Most people just go along with the program because especially with law enforcement there, they don’t want to ruffle any feathers. You can go to the hospital without handcuffs or with handcuffs, you decide.

In the other two jurisdictions, overdose victim transfers to a jail or hospital were less common. In one jurisdiction, overdose victims were generally taken to the local drug detoxification facility and released in 3–5 days. In the other jurisdiction where jail and hospital transfers for overdose victims were uncommon, revived overdose victims were described as often refusing transport to the hospital and were then allowed to walk off the scene on their own recognizance.

Officers in the two jurisdictions where overdose victims are detained following an overdose reversal elaborated on how this practice fits into their on-scene responsibilities and how officer discretion is applied in the following three quotes:

I have definitely arrested the user before because when you get frequent calls on people it’s just like I’m not dealing with this today and the county isn’t going to do anything about it but I’ll lock them up for public intox [ication] or if they have a needle on them.

If [I go] on scene and somebody has had Narcan already administered on them, [I tell] them they can either take advantage of the help or go to jail for public intoxication.

It depends on the situation and officer discretion … But if they [overdose victims] have active warrants they will go to the hospital and then sent to jail. [I] seize contraband as evidence on scene.

### Perspectives about on scene responsibilities and actions

Present across most interviews was a tension about officers having an effective tool to revive someone and save a person’s life in the short term, but no resources to do much else beyond their typical law enforcement duties (i.e., confiscating their drug paraphernalia; arresting them) to help the person in the long term (e.g., connecting them to an accessible substance use treatment). Frustration with not being able to help in the long term was frequently mentioned, and misguided ideologies about how confiscating drug paraphernalia might be helpful were common.

Whenever I am on scene, I go all in. If they have a purse, I go through it. If they have needles, I’m getting rid of them. If you have anything that is there, I’m putting it into evidence. You are not going to keep it. I just feel like that on every call, that helps with them doing it again. I feel like if I take it away from them, then they won’t have it. I say to everyone, ‘There is nothing we can charge you with, I am here to help you.’ But I am going to take it away from them because I am here to help and not contribute to it. In that situation, they are a patient. I would rather put it into evidence [than charge them] because the next time this is going on, if they don’t trust me to start with, then someone might say ‘don’t call the police, my friend got charged the last time we called’ and then that person dies.

Other officers noted that arresting people who have overdosed could be counterproductive in the long term. As one officer noted, ‘We were told that if you get on scene and someone has overdosed to not arrest that person for having paraphernalia to encourage people to call 911.’

Finally, officers who had responded to a high volume of overdose calls described feelings of compassion fatigue and elaborated on how that can impact how officers respond. An example quote is as follows:

Everybody is just a little burned out. You deal with so many overdoses all the time. There for a while overdoses were one of our most common calls [for service]. You would get district-wide probably 10 a day. I personally narcanned three different people in one day just in an 8-hour shift …. I was the guy who was still saying we need to save these people so I would go in [to administer naloxone] when everyone else was saying ‘we can just wait on the medics.”

## Discussion

The most salient finding from our study of law enforcement agency overdose responses is that following an overdose reversal, law enforcement agencies typically force the person who has been revived to either go to a hospital or to jail, or in the case of one jurisdiction, take them to a drug detoxification facility. The policy that people have to go to the hospital reflects the International Association of Chiefs of Police’s (IACP) guidance suggesting that ‘After receiving naloxone, an individual should be transported to a hospital for further medical care’ (International Association of Chiefs of Police, 2017). The IACP guidance notes that jurisdictions need to develop protocols for when a person refuses further treatment or transport to the hospital in accordance with local and federal laws and regulations. Past research has demonstrated that further medical oversight or treatment of an overdose victim post naloxone administration is rarely necessary (Kolinsky et al.*,*^2016^; Levine et al.*,*^2016^; Vilke et al.*,*^2003^; Wampler et al.*,*^2011^; Willman et al.*,*^2017^) though some have noted that those studies did not account for high potency synthetic fentanyl compounds that have been responsible for overdoses in recent years (Glober et al.*,*^2020^) and others have found that refusal to be transported to the hospital post-naloxone administration was associated with subsequent non-fatal overdose (Zozula et al.*,*^2021^). However, syringe services programs provided 702,232 naloxone doses to 230,506 persons in 2018 (Lambdin et al.*,*^2019^), about 17% of which generally end up being used to reverse overdoses by lay persons (Wheeler et al.*,*^2015^). These administrations do not require transfer to hospitals because it is rare that someone needs medical attention post-overdose. Additionally, a safe consumption site in the U.S. recorded 33 overdoses which were all reversed by non-clinical staff and none of them required 911 calls or transfers to medical care (Kral et al.*,*^2020^). Although there are documented cases of people slipping back into overdose after the naloxone wears off, these are very rare events and do not justify taking everyone to a hospital post-overdose. This is important because people who use drugs report having negative experiences in emergency department settings (Meyerson et al., 2021*,* in press; Bergstein et al.*,*^2021^) which can in turn dissuade them from calling 911 for future overdose events (Ellis et al.*,*^2020^). We urge law enforcement departments to reconsider their policies to force overdose victims to the hospital when state or local policies or laws do not mandate this practice. Although there could be some benefits from transporting people who have just overdosed to emergency departments that have robust recovery support, medication-assisted treatment (e.g., buprenorphine induction), and other harm reduction programs (Chen et al.*,*^2020^; Collins et al., 2021; Curran et al.*,*^2021^; D’Onofrio et al., 2015; Herring et al.*,*^2021^), most emergency rooms do not offer culturally competent services for people who have overdosed (Bergstein et al.*,*^2021^; Biancarelli et al.*,*^2019^; Chan Carusone et al.*,*^2019^; Reddy et al.*,*^2021^). However, the continued demonstrated public health efficacy of naloxone “take home programs” (McDonald et al.*,*^2017^) point to how first responder “leave behind” naloxone programs—which involve providing kits on scene to the overdose victim, family, and/or friends of the person who overdosed—could greatly mitigate future overdoses and show early promise for connecting victims to treatment (Scharf et al.*,*^2021^).

Our findings also highlighted that some officers thought that naloxone enables drug use behaviors, which echoes other studies (e.g., Winograd et al.*,*^2020^). Although some officers reported they have been told not to arrest people at the site of an overdose because it could deter future overdose calls for service, officers showed that they have discretion regarding how they respond to overdose incidents. The officers reported that they examine personal effects as part of their responsibilities for resolving the call for service and look to confiscate any drugs or paraphernalia to discourage future drug use, yet rarely find these items on scene. This study suggested that public intoxication and resolving outstanding warrants may be the primary reasons why overdose victims would be taken to a detention facility. This finding supports Deonarine et al.’s (2016) point that it is customary practice for officers to document the names of all present at the overdose scene to determine if there are outstanding warrants.

Our finding that officers force overdose victims to go to jail if they do not consent to be transported to hospitals corroborates other studies (e.g., Carroll et al.*,*^2020^; Lowder et al.*,*^2020^). Carroll and colleagues’ study showed that over one-third of surveyed officers who had administered naloxone reported arresting someone following an overdose reversal (2020) and community-based studies of people who use drugs have also found that overdose victims are often taken to jail (e.g., Park et al.*,*^2019^; Wagner et al.*,*^2019^). For example, a community-based study of people who injected drugs in San Diego found higher proportions of arrest among those who had overdosed in the past 6 months compared to those who had not overdosed (43% vs. 26%, *p* = 0.02; Wagner et al.*,*^2015^). At least one jurisdiction in the U.S. has taken the overdose call for service as a directive to prosecute overdose victims. In February 2020, the Hancock County Indiana prosecutor announced that if police respond to an overdose scene, a search warrant will be issued and officers will investigate for further evidence of illegal drug use, in addition to the illegal drug possession; if evidence is found, the charge would likely be a Level 6 felony, punishable up to 2½ years in jail (Deer, 2020).

All of these punitive responses to an overdose reversal are concerning because they undercut the intentions of GSLs. Officers’ discretionary use of arrest powers for low level offenses like public intoxication threaten public health and could lead to more overdoses. Bohnert et al. (2011) used trend data to show that increased levels of police activity, measured by misdemeanors, was associated with higher overdose mortality after adjusting for overall drug use and demographic characteristics in New York City between 1990 and 1999. More recently, Lowder et al. (2020) compared outcomes following nonfatal overdose where either police or emergency medical services administered naloxone and found that those individuals who received a police response were more likely to be arrested and had more instances of repeat nonfatal overdose two years following dispatch compared to those who had received an EMS response. Research has demonstrated that fear of police harassment, police involvement, and arrest discourages bystanders from calling for help in the future (Deonarine et al.*,*^2016^; Ellis et al.*,*^2020^; Koester et al.*,*^2017^; Latimore and Bergstein 2017; Schneider et al.*,*^2020^; Wagner et al.*,*^2019^). Latimore and Bergstein’s (2017) and Koester et al. (2017) research shows that people who use drugs have myriad reasons for not wanting to call the police, including being fearful of arrest for drug/paraphernalia possession, outstanding warrants, and trespassing as well as fear of losing housing or custody of children, social stigma, and facing violent repercussions from drug suppliers.

The violence that was described by some officers in our study provide another compelling reason why people would not want to call police for an overdose. As noted earlier, an officer described trying to revive unconscious people by kicking them in their ribs, and in another account, an overdose victim was kicked in the face. Although these types of descriptions were in the minority of responses, police violence towards people who use drugs has been documented elsewhere (e.g., Park et al.*,*^2019^). These types of interactions with law enforcement cause irreparable damage to the person who overdosed and the communities in which they live as bystanders bear witness to this violence. These law enforcement responses undermine GSLs and may result in more harm, which has spurred some to call for ceasing police involvement for overdose calls for service (van der Meulen et al.*,*^2021^). Others have called for broader reforms to dispatch laws and practices (e.g., Neusteter et al.*,*^2019^).

Notably, law enforcement agencies serving jurisdictions with high overdose rates are increasingly engaging in programs designed to link people who use drugs with referrals and treatment programs (e.g., Angel programs), deflection or diversion programs, and/or are collaborating with other community partners in co-response overdose programs. Broad reviews of these programs have shown that they vary in their timing, components, services, and follow-up and, as a whole, have not been evaluated (Bagley et al.*,*^2019^; Formica et al.*,*^2021^; Yatsko et al.*,*^2020^) with few exceptions. The Gloucester Police Department’s Angel program was assessed to determine treatment accessibility, participant sobriety, and treatment retention with a 6-month follow up period (Schiff et al.*,*^2017^). This program was found to link participants to short-term detoxification services but was unable to overcome challenges associated with a fragmented treatment system (Schiff et al.*,*^2017^). The Law Enforcement Assisted Diversion (LEAD) program, which is designed to divert people from arrest for certain types of low-level crimes (e.g., drug possession, prostitution) into voluntary treatment, is one of the few deflection programs that has been evaluated, but only in Seattle (Collins et al.*,*^2017^). The nonrandomized controlled evaluation examined LEAD effects on criminal recidivism (i.e., arrests, criminal charges) and found that Seattle LEAD participants had lower rates of arrests and felony charges compared to controls, indicating positive program impacts on recidivism (Collins et al.*,*^2017^).

Some communities are also considering the use of mobile crisis intervention teams that are designed as an alternative to police response for non-violent crises, such as the Crisis Assistance Helping Out On The Streets (CAHOOTS), which began in Eugene, Oregon over 3 decades ago. Although it has been adopted in other cities (e.g., San Francisco), CAHOOTS has never had a process, outcome, or cost-benefit evaluation conducted, either internally or by a third party, to determine its impact or effectiveness. Even though these programs may show early promise in curbing overdoses and connecting people to treatment, rigorous evaluations of these programs are needed to ensure that they are effective and do not have any unintended harmful impacts.

In the near-term, the increasing rate of overdoses in the U.S. mandates an urgent need for law enforcement to reconsider their overdose response policies and practices (Ahmad et al., 2021; American Medical Association, 2020). We call upon law enforcement leadership—including those at the highest levels of leadership (i.e., International Association of Chiefs of Police, National Sheriffs’ Association)—to request agencies to re-examine their policies and procedures concerning search and seizures, warrant reviews, and arrest policies as they specifically relate to overdose calls for service. This is particularly critical since more than 40 states have reported spikes in opioid overdoses since the onset of the ongoing COVID-19 pandemic (American Medical Association, 2020). Moreover, law enforcement leadership may need to consider ways to overcome compassion fatigue (Carroll et al.*,*^2020^), particularly during peak periods of overdose, since it is known that stress and burnout can impact officer performance (Hope 2016).

This study has some potential limitations. The study population of law enforcement officers from the four LEAs was small and respondents were not selected randomly. The study team was reliant on each of the LEA contacts who identified available officers who would be willing to participate. Thus, the responses documented herein do not reflect law enforcement generally or even the law enforcement agencies for which they work. Second, the four diverse agencies selected for this study component were purposively chosen for the characteristics detailed earlier in the methods section and thus are not generalizable to other agencies. However, the goal of qualitative research is not generalizability, but to illuminate the path for further research, which this study has done by providing insights into officers’ perspectives on what happens on scene when they respond to overdose calls for service.

## Conclusion

Law enforcement officers have a unique opportunity to save lives using an easy-to-use public health tool (naloxone). Some jurisdictions are even using the overdose reversal incident as a stepping stone for law enforcement and its partners to connect individuals to treatment and social services (e.g., Bagley et al.*,*^2019^; Formica et al.*,*^2021^). Our findings suggest re-imagining trainings and policies for law enforcement that engender community trust during overdose emergencies are needed. First, we envision policies that strengthen and enhance current GSLs which would preclude law enforcement from conducting searches or arresting people at the scene of an overdose, as these types of changes will make community members more likely to call 911 in future overdose events. Second, determinations about whether to take the person to a hospital should be made by EMS and the person, without involvement of police, and the mere act of having overdosed should under no circumstance be cause for arrest or incarceration. Similarly, EMS systems should re-evaluate their policies to provide clear guidance on the rare situations in which transport to hospital is absolutely needed. Naloxone trainings for law enforcement officers should include information about drug use, people who use drugs, ongoing support for officers who experience fatigue in handling the national overdose crisis, and GSLs.

Finally, we suggest that law enforcement agencies consider asking people with lived experiences from the community to advise them about their policies and practices surrounding their naloxone programs and any other related overdose response programs. Further, we suggest inviting people with lived experiences to be an active part of relevant overdose response trainings, including naloxone training, to answer questions and provide a needed perspective to officers. In addition to their important perspective, the inclusion of people with lived experience to inform law enforcement overdose response policies and practices will also demonstrate the LEA’s commitment to serving *all* of its community members and help build stronger relationships. People with lived experiences could be key in helping police identify innovative, effective community-based solutions that involve trusted local agencies and resources for overdose response, and therefore, require less police involvement so that officers can instead concentrate on serious, violent crimes that threaten public safety.

## Data Availability

The qualitative data collected and analyzed for this study are not publicly available due to confidentiality restrictions and concerns.
